# Subcutaneous Low-Density Foreign Bodies Detection via Grating-Based Multimodal X-ray Imaging

**DOI:** 10.1007/s10278-021-00569-5

**Published:** 2022-01-21

**Authors:** Meifang Yin, Mingzhou Yuan, Kai Deng, Jing Li, Guangya Zhang, Jiayuan Zhu, Weiping Xie, Jun Wu

**Affiliations:** 1 Department of Burn and Plastic Surgery, Department of Wound Repair, Shenzhen Institute of Translational Medicine, The First Affiliated Hospital of Shenzhen University, Second People’s Hospital, ShenzhenShenzhen, 518035 China; 2 Department of Burn Surgery, The First Affiliated Hospital, Sun Yat-Sen University, Guangzhou, 510080 China; 3 Institute of Fluid Physics, Chinese Academy of Engineering Physics, Mianyang, 621999 China

**Keywords:** Grating-based multimodal X-ray imaging, Low-density foreign bodies, Soft tissue, Phase-contrast imaging, Dark-field imaging

## Abstract

Detecting low-density foreign bodies within soft tissues still stands for a serious challenge. Grating-based multimodal X-ray imaging typically has low hardware requirements while simultaneously providing three kinds of imaging information, i.e., absorption, phase-contrast, and dark-field. We aimed to explore the capacity of grating-based multimodal X-ray imaging technology for detecting common foreign bodies within subcutaneous tissues, and to assess the advantages as well as disadvantages of the three kinds of images obtained via grating-based X-ray multimodal technology in relation to diverse kinds of foreign bodies within different tissues. In this study, metal, glass, wood, plastic, graphite, and ceramic foreign bodies were injected into chunks of the pig adipose tissue and chicken thigh muscles. Next, a grating-based multimodal X-ray imaging device developed in our laboratory was used to detect the above foreign bodies within the adipose and muscle tissues. Our results show that grating-based multimodal X-ray imaging clearly revealed the subcutaneous foreign bodies within the adipose and muscle tissues by acquiring complementary absorption, phase-contrast, and dark-field imaging data in a single shot. Grating-based multimodal X-ray imaging has an exciting potential to detect foreign bodies underneath the epidermis.

## Introduction

Foreign bodies penetrations into the skin and other soft tissues following traumata or other accidents are common occurrences that can persistently cause local pain, swelling, abscess, or pseudotumor formation [1]. Basically, the diagnosis of a subcutaneous foreign body includes the patients’ history of trauma, complaints of tingling sensations of foreign bodies, and an instrument-assisted examination of the suspected area [2]. Moreover, it is essential to preoperatively evaluate the length, width, depth, and number of the foreign objects and to mark the involved skin areas to minimize incision length and procedure duration [3]. As children rarely or hardly take the initiative to complain of tingling sensations due to foreign bodies, an imaging exam is especially mandatory for them [4]. The diagnosis and treatment of subcutaneous foreign bodies depend heavily on the imagological examination.

Generally, when a foreign body is suspected, the need often arises to perform exams such as X-ray radiography, ultrasound imaging, computed tomography (CT), and magnetic resonance imaging (MRI) to gain a visual representation of it [5]. According to some reports, the rate of a missed first diagnosis of a subcutaneously placed foreign body reaches up to 38% [6]. Commonly, the foreign bodies materials are metal, glass, wood, plastic, graphite, and ceramic [7, 8]. In general, X-ray and CT pictures can reveal the metal, glass, and ceramic foreign bodies, but the diagnostic rate of wooden foreign bodies is only 15%, which is extremely unfavorable from the standpoint of further surgical treatments [6]. Ultrasound imaging relies heavily on the skill and practice of the operator. A recent meta-analysis found that ultrasound imaging has only a 72% sensitivity in relation to foreign bodies identification in soft tissues [9]. According to reports, ultrasound imaging can hardly detect soft-tissue foreign bodies with cross-section lengths of less than 2 mm [10]. Although MRI often has a high resolution, an iron foreign body will both be dangerous and create a huge observational artifact [11]. Moreover, MRI is not instrumental in displaying foreign bodies with low water contents. In addition, for the examination of subcutaneous foreign bodies, MRI has no obvious superiority due to its slow imaging speed and expensive equipment [11]. The just mentioned facts prompted us to develop novel more helpful techniques for the diagnosis of subcutaneous foreign bodies.

X-ray phase-contrast imaging was developed in the 1990s, while grating-based multimodal X-ray imaging, which has minimal hardware requirements, was established in 2003 [12]. The latter utilizes the samples’ differences in X-ray absorption, capacity, electron density distribution, and small-angle scattering to simultaneously obtain three kinds of image information related to absorption, phase-contrast, and dark-field [13]. Standard X-ray absorption imaging would not show any contrast between different soft tissues, because of their relatively homogenous X-ray attenuation coefficient [14]. Phase-contrast and dark-field imaging are complementary. It was reported that X-ray phase-contrast imaging appears thousand times more sensitive to soft tissue than traditional absorption imaging [15], and so grating-based multimodal X-ray imaging shows great potential in medical application. At present, one or more technologies of grating-based multimodal X-ray imaging have been reported to be used in the preliminary research in diagnosis of breast [16], hepatobiliary [17], cartilage [18], lung [19], angiocarpy [20], and nervous system diseases [21]. Due to the large difference in the nature of X-ray scattering between common foreign materials and human soft tissues [22, 23], grating-based multimodal X-ray imaging technology is expected to effectively detect subcutaneous foreign bodies [24]. It would be of great value if a single device could detect various of foreign bodies that are common in clinical trauma. The purposes of our present work were (i) exploring the capacity of grating-based multimodal X-ray imaging technology for detecting common foreign bodies within subcutaneous tissues and (ii) assessing the advantages and disadvantages of the three kinds of images obtained via grating-based X-ray multimodal technology in relation to diverse kinds of foreign bodies within different tissues.

## Materials and Methods

### Equipment and Parameters

**Fig. 1:**
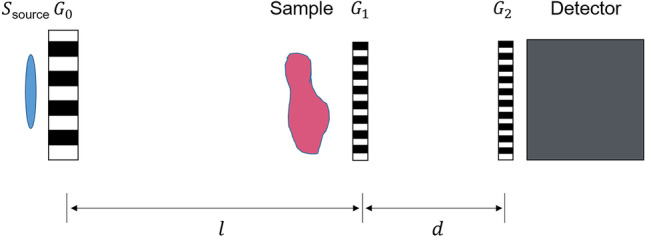
Schematic representation of an X-ray grating interferometer

This device uses an imaging technique previously devised by Pfeiffer et al. [25]. Three X-ray transmission gratings arranged between the conventional clinical X-ray source and the X-ray detector serve to enable the recording of the physical images (Fig. 1). The first grating downstream the sample (G1) generates a periodic intensity pattern. The latter must be passed through another absorption grating (G2), since otherwise it is too dense for a direct observation by a clinical X-ray detector. The analysis of the periodic intensity pattern is implemented through a phase-stepping approach, which means translating one of the gratings and acquiring images at intermediate positions. The thus obtained curve, typically named stepping curve, corresponds to the registered intensity in each pixel as a function of the positions. The visibility of this stepping curve is a key parameter for the X-ray grating interferometer. The last grating (G0) is found next to the X-ray source, splitting the X-ray spot into a series of line sources to improve the lateral coherence.

The X-ray source, a clinical tungsten-target X-ray tube with a focal spot size of 400 μm, was run at 55 kV voltage and 8.5 mA tube current. The choice of the acceleration voltage was a compromise between the achievable visibility (over 20%) and a reasonable transmission rate. A 7-step phase was used in the periodic intensity pattern analysis, with a 50 s exposure for each step. A digital X-ray imager with 127 μm pixel size (Varian PaxScan 1313DX) was used for image acquisition. The distances between gratings and the parameters of the gratings are listed in the following tables (Tables 1 and 2) [26].

**Table 1:** Distances between gratings

Components	Distance
Source grating (G0) − Phase grating (G1)	2156 mm
Phase grating (G1) − Analyzer grating (G2)	308 mm

**Table 2:** Parameters of the gratings

	G0	G1	G2
Material	Au	Au	Au
Period (μm)	21	5.25	3
Height of bars (μm)	85	6.29	91
Duty cycle	0.52	0.54	0.53

### Material Preparation

We utilized the following foreign objects: (i) metal intravenous needles 0.45*0.45*5 mm in size; (ii) fragmented glass pieces 3*5*0.8 mm in size; (iii) bamboo sticks about 1*5*1 mm in size; (iv) polyethylene plastic sheets about 3*5*0.2 mm in size; (v) graphite pencil tips about 1*4*1 mm in size; (vi) ceramic chips about 3*5*2 mm in size. All of them were injected to mimic soft-tissue damage due to foreign bodies.

**Fig. 2:**
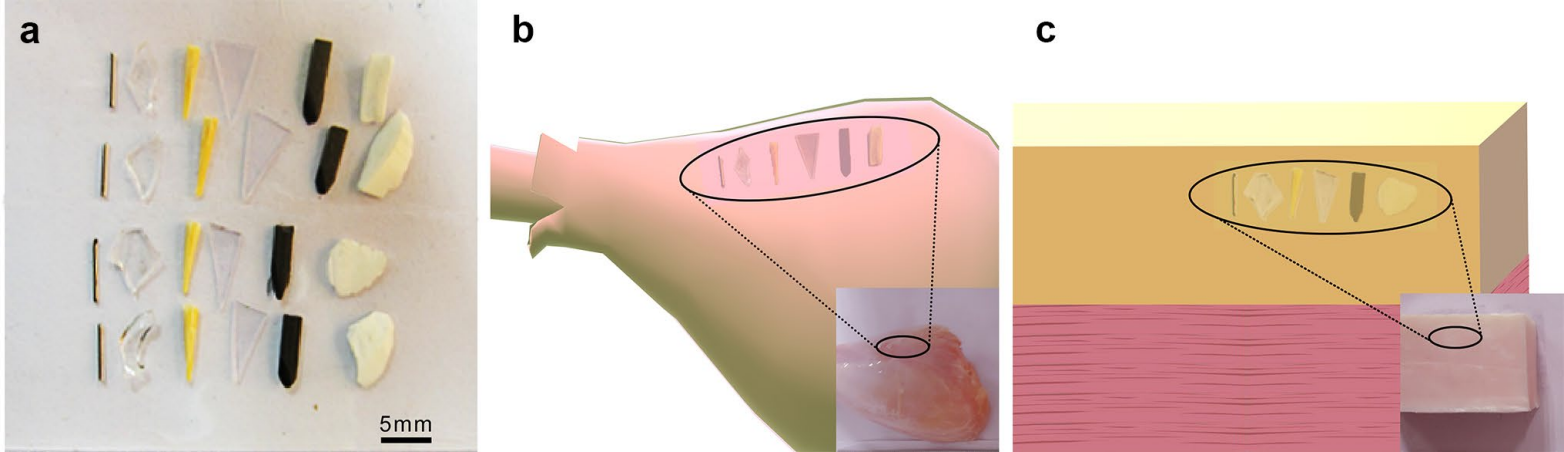
Experimental materials.** a** Foreign bodies made of metal, glass, wood, plastic, graphite, and ceramic (respectively). **b** Puncture of foreign bodies into a skinned chicken thigh. **c** Puncture of foreign bodies into a chunk of pig adipose tissue

To assess whether such foreign bodies, which are commonly encountered in life, were detectable within layers of fat, large chunks of pig adipose tissue were used to closely resemble the adipose layer of human subcutaneous tissues. To evaluate the feasibility of detecting foreign bodies in muscle tissue and to avoid unwanted effects from the dried unevenly exposed muscle fibers, we used freshly skinned chicken thighs. As shown in Fig. 2b and c, the abovementioned objects were inoculated in turn into pig adipose tissue and chicken muscle tissue.

### Imaging Acquisition

Chicken muscle and pig adipose tissues were fixed on frames and next placed into the grating-based multimodal X-ray instrument. After the imaging procedure was completed, the chicken muscle and pig adipose tissues were scanned in a CT (SOMATOM Definition Flash, SIEMEMS, Germany; parameter settings: slice: 0.75 mm; tilt: 0 mm; WL: 450; WW: 1500; kV: 100; mA: 68) and in an MRI system (Achieva 1.5 T, PHILIPS, Netherlands; parameter settings: slice: 1 mm; WL: 750; WW: 3500). Prior to the MRI scan, the metal tip and pen core foreign bodies were taken out because of their metal contents that would have generated significant artifacts thereby affecting the observation of the surrounding objects. Next, echograph examinations were performed via an ultrasound machine (SuperSonic Imagine, Aix en Provence, France). A linear array transducer having a 15-MHz frequency bandwidth was used for all the conventional ultrasound examinations.

### Data Processing

#### Signal in Absorption and Dark-Field Imaging

Concerning the absorption, phase-contrast, and dark-field imaging results, the pixel line segments of interest were selected, and the signal of each pixel of the same segments was acquired from the source files and plotted as a distance-intensity curve according to the position sequence [27]. In order to quantify the visibility of foreign objects, the signal value of various foreign bodies was extracted and divided by the basal signal of adjacent tissues to obtain the “signal ratio” value. A significant difference between the signal ratio value and 1 would indicate that there is a significant difference in signal intensity between the foreign body and the surrounding tissue, which means foreign bodies are more easily observed.

#### Ratio Value

Since the signals of both the absorption and dark-field are linearly related to the sample thickness, their ratio value allows to eliminate the unknown thickness value parameter [28]. Since the dark-field signal usually increases at the boundary of the object, while the absorption signal generally decreases at the boundary, their ratio is conducive to highlighting the signals of the outlines of subcutaneous foreign bodies [29]. The ratio value (named R′ value) of the foreign body in vitro was calculated by dividing the foreign body dark-field results by the absorption imaging results. The R′ values of the fat and muscle tissue image results could also be calculated by using the same algorithm.

#### Pseudocolor Maps

The results of absorption, phase-contrast, and dark-field imaging were respectively converted into the primary 256-level colors red, blue, and green. Next, the corresponding images were merged to obtain pseudo-color maps.

#### Statistical Analysis

Statistical tests were performed using SPSS software, version 22 (IBM, Armonk, US), with significance defined as *P* < 0.05. In the comparison of R′ data of various foreign bodies and soft tissues, Games-Howell one-way ANOVA was used for the data conforming to normal distribution, while Kruskal–Wallis rank sum test was used for the data not conforming to normal distribution.

## Results

### In Vitro Imaging

**Fig. 3:**
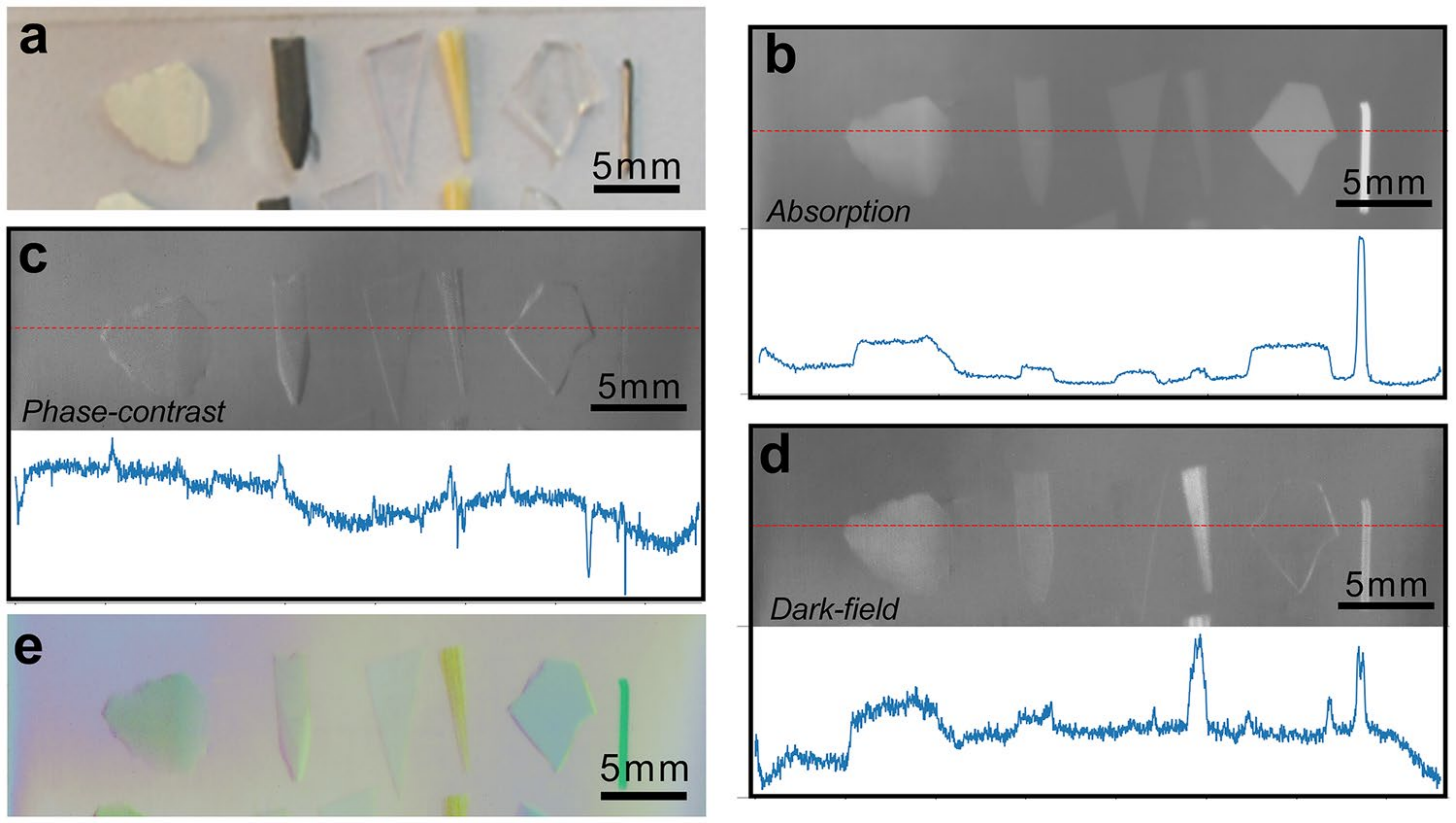
Results of common foreign bodies grating-based X-ray multimodal imaging in vitro and the original signal curves corresponding to each position in the cross-section displayed in the line segment. **a** Photograph of isolated common foreign bodies. **b** The absorption image of **a**. **c** The phase-contrast image of **a**. **d** The dark-field image of **a**.** e** The pseudo-color image of **a**

The absorption, phase-contrast, and dark-field data in vitro clearly and correctly revealed the morphology and size of these foreign bodies. By extracting the signal intensity value of each pixel of the results’ line segments, the distance-intensity curves could be plotted. As the curves show (Fig. 3b–d), the values of the absorption imaging signals are high at the location of foreign bodies, while the phase imaging signals are high at the boundaries of foreign bodies. In other words, absorption imaging directly shows the objects, while the phase-contrast imaging reveals the boundaries of the same objects. The dark-field results could directly detect the metal, wood, graphite, and ceramic foreign bodies, but did only unveil the boundaries of glass and plastic foreign objects.

### Foreign Bodies in Adipose and Muscle Tissues

**Fig. 4:**
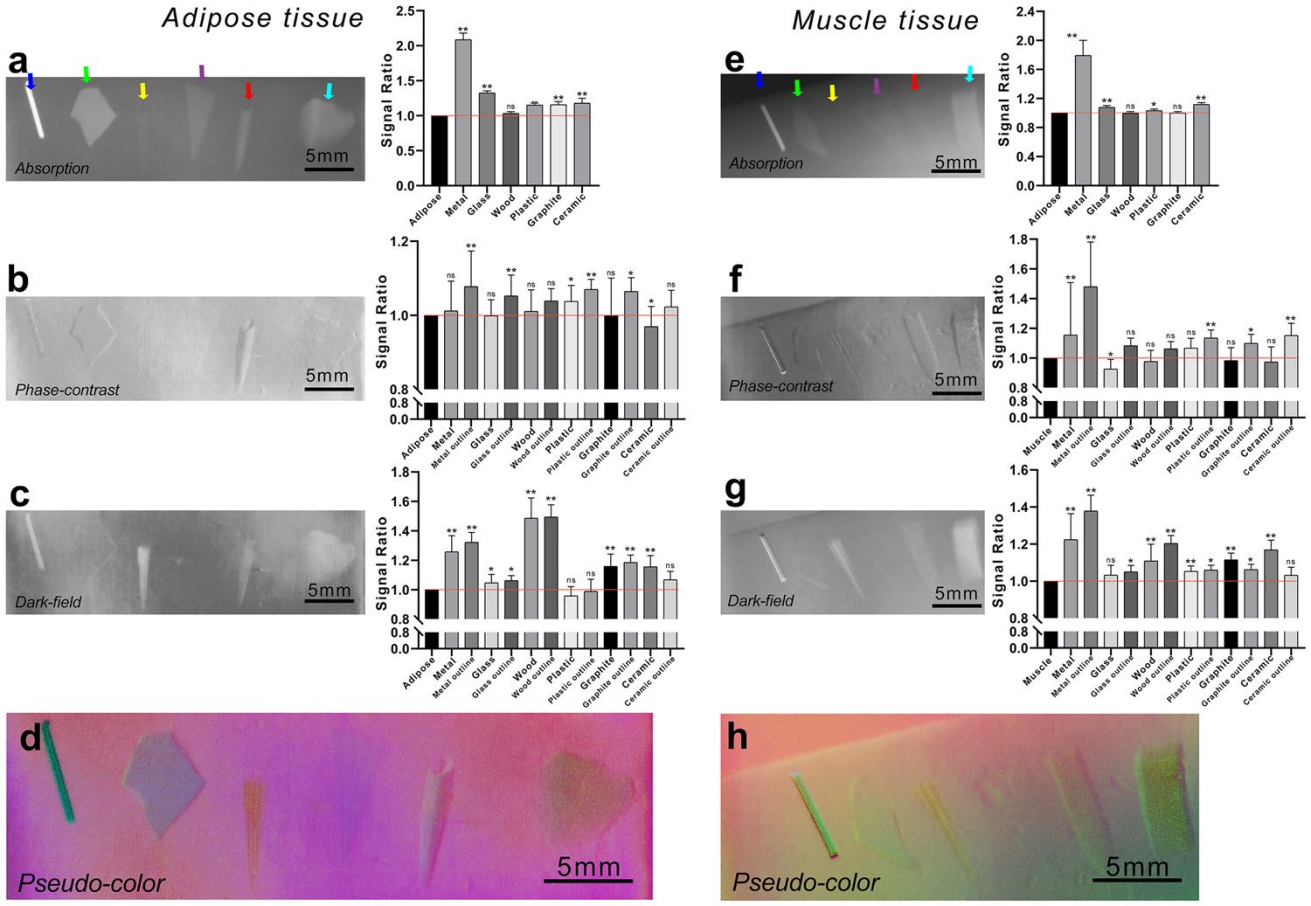
Grating-based X-ray multimodal imaging results of foreign bodies within the adipose and muscle tissues. Objects within adipose tissue and the corresponding signal ratio histograms, **a** absorption image, **b** phase-contrast image, **c** dark-field image, and **d** pseudo-color image. Objects within muscle tissue and the corresponding signal ratio histograms, **e** absorption image, **f** phase-contrast image, **g** dark-field image, and **h** pseudo-color image. (The colored arrows indicate the locations of foreign objects. Blue arrows point to the position of the metal needle; the green arrows to the position of the glass object; the yellow arrows to the position of the wooden object; the purple arrows to the position of the plastic object; the red arrows to the position of the graphite object; and the cyan arrow to the position of the ceramic object.) ns indicates *P* > 0.05, * indicates *P* < 0.05, ** indicates *P* < 0.01

Regarding the adipose tissue, the absorption imaging result was a clear display of the foreign objects except for the wooden sticks. The signal ratio of the wooden stick was close to 1, indicating that there was no difference between the signal value of them and the adjacent adipose tissues (Fig. 4a). The signals of boundary contours of the foreign bodies were higher than the adipose tissue in phase-contrast imaging results except for wooden stick (Fig. 4b). However, in dark-field imaging, wooden foreign objects gave a clear and strong signal. Only the plastic did not get a strong signal in the dark-field imaging (Fig. 4c).

Regarding the muscle tissue, it was extremely difficult to recognize the wood and graphite foreign bodies from the absorption imaging results due to the similar signal strength between them and muscle tissue (Fig. 4e). The phase-contrast imaging results allowed to distinguish from the muscle tissue all six types of foreign bodies, among which the 0.2 mm plastic sheet was the most difficult to detect although part of its boundary contour was reinforced (Fig. 4f). The dark-field results of the muscle tissue were similar to those of the adipose layer, while it was difficult to identify the plastic sheets in both tissues (Fig. 4g).

By merging the three imaging results into pseudo-color images, all six foreign bodies could be clearly identified in the adipose tissue. On the other hand, in the muscle tissue, five foreign bodies could be clearly detected, while the plastic sheets were indistinct (Fig. 4d and h).

### Results of CT, MRI, and Ultrasound Imaging

**Fig. 5:**
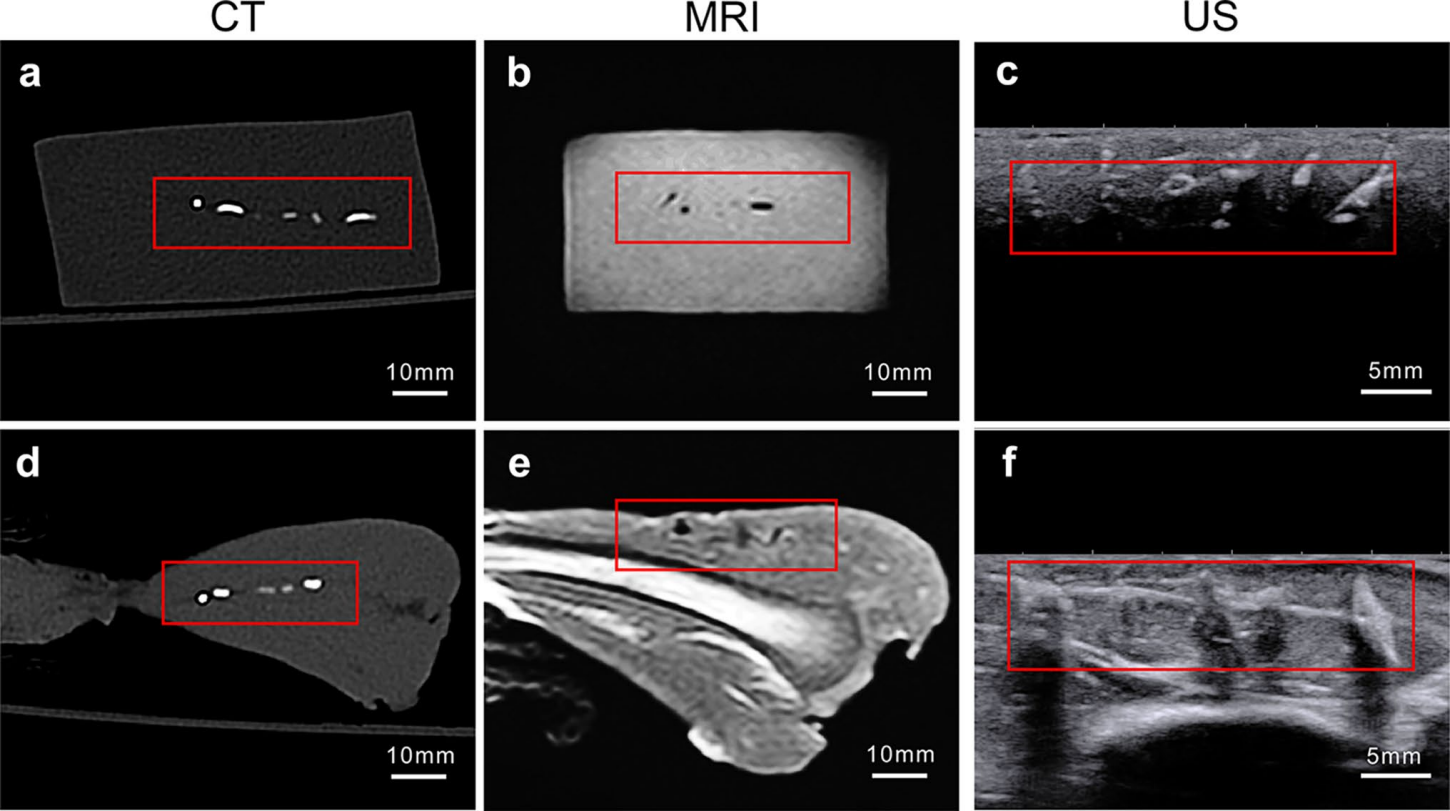
Results of common imaging diagnostic methods. **a**, **d** CT scan results of metal, glass, wood, plastic, graphite, and ceramic foreign bodies in adipose and muscle tissues. **b**, **e** MRI scan results of glass, wood, plastic, and ceramic foreign bodies in adipose and muscle tissues. **c**, **f** Ultrasound imaging results of metal, glass, wood, plastic, graphite, and ceramic foreign bodies in adipose and muscle tissues. The red box indicates the location of the foreign bodies

*CT*: The CT scan results allowed to detect the six kinds of foreign bodies in the adipose tissue; and except for the wooden objects, all the others were obvious. In the muscle, only five types of foreign bodies could be recognized, while the wooden foreign bodies remained undetected (Fig. 5a and d). *MRI*: It was hard to detect glass, wood, plastic, or ceramic foreign bodies via MRI imaging. Because these four kinds of foreign bodies give out weak MRI signals, in adipose and muscle tissues, they can only be indirectly deduced from the morphology of the surrounding soft tissues. This makes detecting such foreign bodies more difficult (Fig. 5b and e). *US*: The sinus tracts caused by the injection of each kind of foreign body could be seen via ultrasound imaging because the air in their interiors was strongly echogenic. But ultrasound imaging could not reveal the full shape of foreign objects both in adipose and muscle tissues. This happened because the stronger echoes of the foreign bodies outer parts that were closer to the probe blocked the imaging of their inner areas. Moreover, it was hard to detect via ultrasound imaging foreign bodies, such as needles and toothpicks, whose cross-sectional areas were very small in comparison with the probe’s area (Fig. 5c and f). In general, the techniques mentioned above are difficult to distinguish all kinds of foreign objects in the sample in a single shot.

### R′ Values Reconstructed Image

**Fig. 6:**
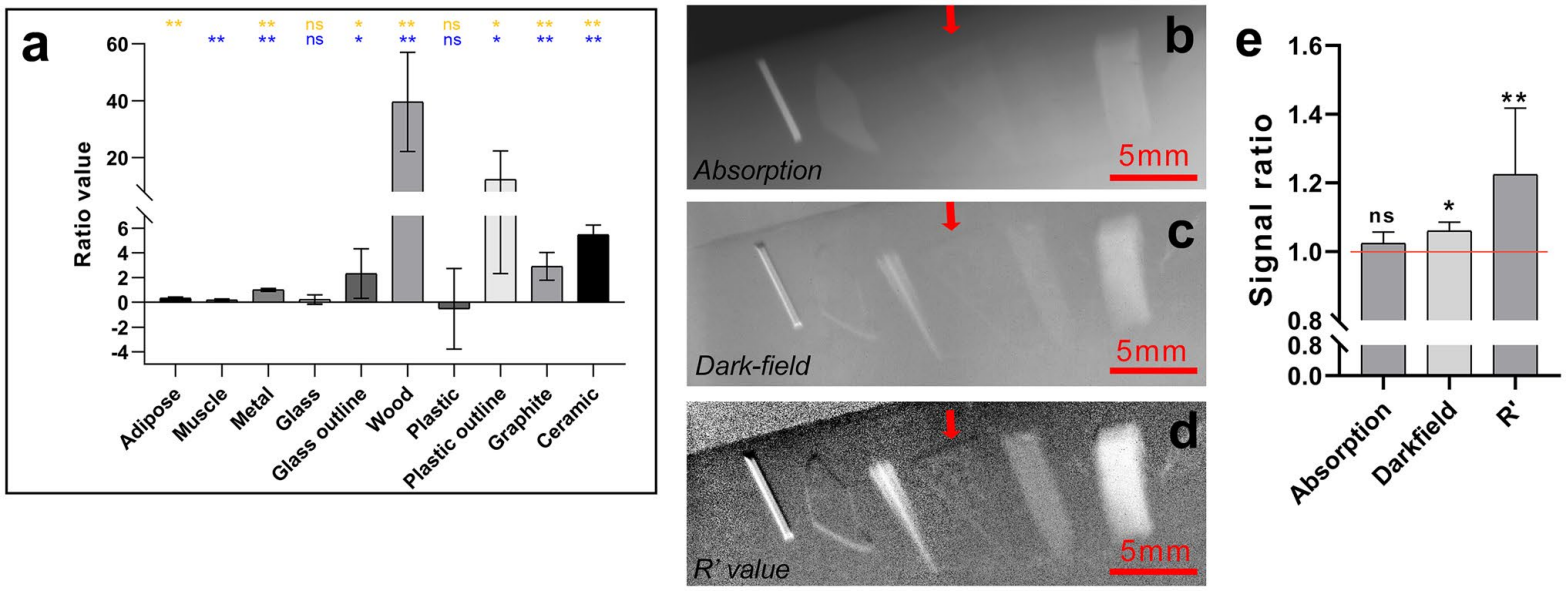
R′ values calculated by dividing the dark-field results by the absorption results. **a** Histogram of the R′ values for different tissues and various foreign bodies. The blue asterisks at the top of the bar chart indicate a significant difference between the R′ values of adipose, and the yellow indicate a significant difference with the R′ values of muscle tissue. **b** Absorption picture of foreign bodies within muscle tissue. **c** Dark-field image of the same foreign bodies within muscle tissue. **d** The dark-field values of the same foreign bodies in the muscle tissue were divided by the absorption values and the results reconstructed as image. The red arrow points to the position of the practically undetectable plastic sheet. **e** Signal ratio of outline of plastic within muscle tissue in absorption, dark-field, and R′ imaging results. (ns indicates *P* > 0.05, * indicates *P* < 0.05, ** indicates *P* < 0.01)

By dividing the signals of every pixel from the dark-field results by the signals of the same pixel from the absorption results, the upshot removed the influence of thickness to some extent, and the obtained value (named R′ value) could stand for the essential property of the material. As could be seen in a comparison of the R′ values of different tissues and various foreign bodies (Fig. 6a), the R′ values of metal, wood, graphite, and ceramics were several folds higher than those proper of soft tissues. Though neither glass nor plastic differs significantly from soft tissue, the R′ values of glass outline and plastic outline were significantly (*P* < 0.05) differed from soft tissues. Among them, under the present hardware conditions, it was difficult to directly figure out the type of foreign bodies by calculating the R′ values of the objects.

The imaging of plastic foreign bodies within muscle tissue was significantly improved when the corresponding dark-field values were divided by the absorption values and the corresponding R′ values reconstructed as images (Fig. 6d). It is worth noting that the average of R′ values of plastic outline were 10 times higher than the R′ value of the muscle tissue. Therefore, the plastic sheet, which was difficult to be clearly detected by the absorption and dark-field results (Fig. 6b and c), could be easily detected within the muscle tissue in the image reconstructed according to the R′ values. It can be seen from the signal ratio in absorption, dark-field, and R′ imaging results that the R′ value processing can greatly enhance the recognizability of the plastic (Fig. 6e).

## Discussion

As compared with traditional absorption X-ray imaging, grating-based multimodal X-ray imaging could simultaneously gain information about three aspects of the foreign bodies in the tissue lesions: i.e., absorption capacity, electron density distribution, and small-angle scattering. Therefore, this approach allows a more extended detection of common foreign bodies. As our results showed, detecting soft foreign bodies within muscles via absorption or CT imaging was more difficult than within the adipose tissue, because muscles’ density is closer to that proper of common foreign soft bodies. Although wood and graphite bodies were difficult to distinguish from muscle via absorption imaging, they could be clearly discovered via phase-contrast and dark-field imaging. Moreover, when the results of absorption, phase-contrast, and dark-field imaging were superimposed, the six kinds of subcutaneous foreign bodies presently investigated could be clearly distinguished. The three results directly obtained via grating-based multimodal X-ray imaging, allowed to identify only five kinds of foreign bodies, excepting plastic sheets, within the muscle tissue. But by reconstructing the corresponding R′ values as a single image, the plastic sheet could also be clearly detected within the muscle tissue.

The reason why wooden and graphite foreign objects could be identified via phase-contrast and dark-field imaging while they were hardly spotted via absorption imaging, might be laid on the different mechanisms generating image contrasts. As it is well known, the absorption imaging results derive from the attenuation of the X-rays passing through the samples. As the attenuation coefficient is affected by the tissues’ densities, the absorption images can identify the boundaries between tissues or materials having different densities (such as bones and muscles), but usually fail to do so when the densities are similar [23]. The phase-contrast and dark-field imaging rely instead on refraction and ultra-small-angle X-ray scattering (USAXS), respectively [30]. For a Talbot-Lau grating interferometer, the phase-contrast resulting from the X-ray refraction is proportional to the gradients of the total amount of phase change (or the line-integrated electron density) [25, 31]. The total amount of phase change is hundreds of times larger than that of attenuation, and the gradient usually peaks even at boundaries between tissues or materials having similar densities [32]. These two factors lead to the contrast enhancement of boundaries on the phase images and thus improve the distinction between soft tissues (e.g., adipose, muscle), or soft tissues and materials with similar densities (e.g., wood, glass, and plastic). As for the dark-field contrast, the intensity of USAXS is related to the electron-density inhomogeneities of samples at micro scale, which usually maximizes around boundaries [29]. In addition, the intensity of USAXS also keeps a high value for materials with a directional microstructure (e.g., the wood fibers making sticks) [24]. This explains why both the boundary and the inside area of the wooden sticks are bright on the dark-field images.

The typical features of grating-based multimodal X-ray imaging are a low hardware requirement and the ability to simultaneously gain three kinds of imaging information, i.e., absorption, phase-contrast, and dark-field, which confers a great potential in screening soft tissue lesions. It was discovered that the R′ values of different materials greatly differ when using grating-based multimodal X-ray imaging. It is necessary to notice that the three different physical signals acquired by X-ray interferometer have distinct spectrum characteristic, which have not been thoroughly investigated. As a result, the quantitative analysis of the R value needs much further researches, although the indicative results offered enough support to medical applications. Therefore, more advanced algorithms could broaden its range of application even further. At present, there have been many reports of its implementation for diseases affecting cartilages, vessels, breasts, lungs, central nervous system, and so on [33]. In addition, as the results of the present study show, grating-based multimodal X-ray imaging has a remarkably high capability of identifying soft components. Therefore, it would be helpful to widely use it for the diagnosis of soft tissue and mixed soft tissue-gas lesions. In addition to detecting subcutaneous soft foreign bodies, it may also be of service in the diagnosis of tissue fibrosis, sinus tracts, gas gangrene, and other diseases. Here, we would like to stress that it allows to gain crucial information not obtainable via other techniques allowing to study disease evolution, as well as to perfect/check advanced new therapies.

Of course, this technology will undergo improvements in the future. First, concerning physical engineering, we need to increase the field of view, improve the resolution, and assess the amounts of radiations needed for the imaging of different sites to reduce unnecessary exposure. And we require to increase the total amount of light intensity and the visibility index of the system to reduce the noise in the phase-contrast and dark-field images. Second, in terms of data processing, we need to try different image enhancement and data processing methods to achieve clearer pictures. Third, with more mature image fusion algorithm, quantitative analysis of different objects is needed for more rigorous research. Fourth, regarding disease detection, we require applying it to a wider range of animal disease models to widen the field of clinical applications.

## Conclusions

In conclusion, grating-based multimodal X-ray imaging can at the same time successfully detect both high- and low-density foreign bodies under the skin. Therefore, it is a very promising approach to the medical diagnosis of subcutaneous foreign bodies. It is to be hoped that soon X-ray detection equipment using this technology will supplement the existing standard X-ray imaging equipment, thus making possible a further leap in medical imaging diagnostic technology.

## Data Availability

The data that support the findings of this study are available upon request from the corresponding authors.
